# 

**Published:** 1904-01-16

**Authors:** 


					THE HOSPITAL. "The standard of highest purity."?Lancet. January 16th, 1904.
CADBURY'S U CADBURY'S
COCOA JL MM JQL. COCOA
A PERFECT FOOD." NO DRUGS OR ALKALIES USED.
taoir Wectzrh Infirmary
No. 903. Yol. XXXV. [^|?p?] Saturday, Jan. 16th, 1904. [Sabscsr4Ptp.?270.erm3'] PRICE THREEPENCE.

				

